# Tyro3 Contributes to Retinal Ganglion Cell Function, Survival and Dendritic Density in the Mouse Retina

**DOI:** 10.3389/fnins.2020.00840

**Published:** 2020-08-14

**Authors:** Farrah Blades, Vickie H. Y. Wong, Christine T. O. Nguyen, Bang V. Bui, Trevor J. Kilpatrick, Michele D. Binder

**Affiliations:** ^1^The Florey Institute of Neuroscience and Mental Health, University of Melbourne, Parkville, VIC, Australia; ^2^Department of Optometry and Vision Sciences, University of Melbourne, Parkville, VIC, Australia; ^3^Department of Anatomy and Neuroscience, University of Melbourne, Parkville, VIC, Australia

**Keywords:** TAM receptor, receptor tyrosine kinases, electroretinogram, optical coherence tomography, dendrites, inner plexiform layer

## Abstract

Retinal ganglion cells (RGCs) are the only output neurons of the vertebrate retina, integrating signals from other retinal neurons and transmitting information to the visual centers of the brain. The death of RGCs is a common outcome in many optic neuropathies, such as glaucoma, demyelinating optic neuritis and ischemic optic neuropathy, resulting in visual defects and blindness. There are currently no therapies in clinical use which can prevent RGC death in optic neuropathies; therefore, the identification of new targets for supporting RGC survival is crucial in the development of novel treatments for eye diseases. In this study we identify that the receptor tyrosine kinase, Tyro3, is critical for normal neuronal function in the adult mouse retina. The loss of Tyro3 results in a reduction in photoreceptor and RGC function as measured using electroretinography. The reduction in RGC function was associated with a thinner retinal nerve fiber layer and fewer RGCs. In the central retina, independent of the loss of RGCs, Tyro3 deficiency resulted in a dramatic reduction in the number of RGC dendrites in the inner plexiform layer. Our results show that Tyro3 has a novel, previously unidentified role in retinal function, RGC survival and RGC morphology. The Tyro3 pathway could therefore provide an alternative, targetable pathway for RGC protective therapeutics.

## Introduction

Retinal ganglion cells (RGCs) are the earliest neurons to arise in the retina. RGCs are produced from a common retinal progenitor cell, from which all retinal neurons ultimately arise (Turner and Cepko, 1987; Turner et al., 1990). Once differentiated from retinal progenitor cells, post-mitotic RGCs migrate to their final location in the ganglion cell layer (GCL) of the retina. The axons of all RGCs exit the eye via the optic nerve head, where they bundle to form the optic nerve, ultimately finding targets within the visual centers of the brain. Vertical pathway neurons within the eye are glutamatergic, RGC dendrites within this pathway arborize in the inner plexiform layer (IPL) of the retina, where they synapse with bipolar cells (reviewed in Joselevitch, 2008). One remarkable feature of RGC information processing is the functional stratification of RGC dendrites within the IPL (Famiglietti and Kolb, 1976), whereby the dendrites of ON-RGCs, which respond to incrementing light, stratify within the inner IPL, while the OFF-RGCs, which respond to decrementing light, stratify within the outer IPL (Famiglietti and Kolb, 1976; Nelson et al., 1978). In addition to the functional stratification of RGCs, there are around 40 known subpopulations of RGCs based on morphology and genetic profile (reviewed in Sanes and Masland, 2015).

The death of RGCs is a common outcome in many optic neuropathies, such as glaucoma, demyelinating optic neuritis and ischemic optic neuropathy, resulting in visual defects and blindness (Khatib and Martin, 2017). RGC vulnerability to disease is type-dependent (Christensen et al., 2019), for example, OFF-RGCs are more susceptible than ON-RGCs in an experimental murine model of glaucoma (Della Santina et al., 2013) and an optic nerve crush model (Puyang et al., 2017). Currently, there are no clinically approved therapeutics to prevent RGC death in optic neuropathies; making the identification of new targets to support RGC survival invaluable. A better understanding of the molecular pathways involved in normal RGC development, function and survival could provide novel insights and targets for therapeutic development.

Tyro3 is a member of the TAM family, a unique subfamily of receptor tyrosine kinases which also includes Mertk and Axl. The TAM family of receptors play essential roles in phagocytosis, cell survival and immune homeostasis (reviewed in Tondo et al., 2019). The TAM receptors are activated by two closely related ligands, Pros1 and Gas6. Interestingly, there is a delineation of ligand-receptor interactions, such that Axl is exclusively activated by Gas6, whereas Tyro3 and Mertk are activated by both ligands (Lew et al., 2014). It has been shown that the loss of any single TAM receptor has subtle phenotypic effects, with more overt phenotypes arising from double or triple receptor knockouts (Lu and Lemke, 2001). Tyro3 is the most widely expressed TAM receptor in the brain, with highest expression in neurons of the cortex and hippocampus (Prieto et al., 2007; Pierce et al., 2008; Kim et al., 2017). Using a mouse model of Tyro3 deletion, we and others have previously identified crucial roles for Tyro3 in CNS myelination (Akkermann et al., 2017; Blades et al., 2018), neuronal survival and migration both *in vitro* (Prieto et al., 2007) and *in vivo* (Pierce et al., 2008; Kim et al., 2017). In addition, Tyro3 is expressed in different compartments within neurons, including in the cell body, axons and dendrites (Prieto et al., 2007). In the latter, Tyro3 was shown to partially overlap with expression of postsynaptic density protein-95 (PSD-95), where it was hypothesized to be involved in activity dependent neuronal signaling and long-term potentiation (Prieto et al., 2007).

In the mammalian retina, Tyro3 and Mertk are expressed by the retinal pigment epithelium (RPE) and are critical for photoreceptor disc phagocytosis (Prasad et al., 2006). Furthermore, loss of function mutations in *MERTK* are associated with the development of retinitis pigmentosa in humans (reviewed in Parinot and Nandrot, 2016) and retinal degeneration in rodents (D’Cruz et al., 2000). However, this phenotype can be rescued in mice by enrichment of *Tyro3* (Vollrath et al., 2015). Tyro3 has also been shown to be expressed in a subset of rat RGCs and to some degree across all RGC populations in the mouse (Lindqvist et al., 2010; Rheaume et al., 2018). However, the function of the Tyro3 receptor in RGCs has not been explored.

In this study we investigated the role of Tyro3 on the structure and function of the retina, with a focus on RGCs. We performed full-field electroretinography to assess retinal function, as well as optical coherence tomography (OCT) and immunohistochemistry to assess retinal structure in Tyro3 receptor knock-out mice. We show that Tyro3 deficiency results in impaired function of photoreceptors, bipolar cells and RGCs. The decrease in RGC function was associated with a loss of RGCs and a concomitant thinning of the retinal nerve fiber layer. We identify, in the central retina, that the loss of Tyro3 results in fewer dendrites within the ON-RGC layer of the IPL, in a manner at least partially independent of the loss of RGCs. These findings suggest that Tyro3 plays a newly identified role within the mouse retina and is crucial for dendritic morphogenesis of RGCs.

## Materials and Methods

### Mice

Tyro3^–/–^ mice were fully backcrossed onto C57Bl/6 and maintained in a specific-pathogen-free environment during breeding and experimentation. The Tyro3^–/–^ line was a kind gift of Prof. Greg Lemke (Salk Institute of Biological Studies, La Jolla, CA; Lu et al., 1999). Animal experiments were conducted within the guidelines of our institutional Animal Ethics Committee and in accordance with the National Health and Medical Research Council Guidelines. The experimental cohorts consisted of Tyro3^–/–^ mice (denoted as Tyro3 KO) and Tyro3^+/+^ wild-type littermates (denoted as WT) as controls; cohorts were sex and age balanced.

### Electroretinography (ERG) and Analysis

Dark-adapted, full-field ERGs were used to examine *in vivo* retinal function as previously described (Nguyen et al., 2016; Zhao et al., 2017). Eight-week old littermate controls (*n* = 10) and Tyro3 KO mice (*n* = 10) were acclimatized to the experimental facility for 1 week prior to experimentation. At 9-weeks of age, immediately prior to ERG measurements mice were dark-adapted overnight (>12 h). During the procedure, light exposure was kept at a minimum, using a dim red light to aid animal preparation and set-up. Minimizing white light exposure allows for RGC-specific scotopic threshold response (STR) measurements (Bui and Fortune, 2004). Mice were sedated using ketamine: xylazine anesthesia. Corneal anesthesia (0.5% proxymetacaine, Alcaine^®^, Alcon, Geneva, Switzerland) and mydriatic (1% tropicamide, Mydriacyl^®^, Alcon) were topically applied and animals placed on a heated platform (37°C) for the duration of the experiment. A pair of custom-made silver chloride electrodes (99.9%, A&E Metal Merchants, Marrickville, Australia) were placed on each eye, with an active loop and inactive ring-shaped electrodes (connected to platinum leads, F-E-30, Grass Telefactor, West Warwick, RI) placed on the central cornea and sclera, respectively. A stainless-steel needle electrode (F-E2-30, Grass Telefactor) was inserted subcutaneously into the tail to ground the electrical current. A small amount of gel lubricant was placed on the electrode to maintain corneal hydration and conductance. Light stimuli were delivered to both eyes across a range of light levels (-5.53 to 2.07 log cd⋅s/m^2^) by an array of 8 white light emitting diodes (LEDs, 8 Watt Luxeon LED, Philips Lumileds Lighting Company, San Jose, United States) and a single dim LED (0.1 Watt Luxeon LED, Philips Lumileds Lighting Company) embedded inside a Ganzfeld sphere (Photometric Solutions International, Oakleigh, Australia). ERG signals were acquired from both eyes simultaneously using Scope^TM^ software (ADInstruments Pty Ltd., Bella Vista, Australia) at a 4-kHz sampling rate with pre-amplifier (P511, Grass Telefactor) and hardware band-pass filter settings of 0.3–1000 Hz (-3 dB). Signals were digitized (ML785 Powerlab 8SP, ADInstruments) and saved for *post hoc* processing.

Components of the ERG waveform reflect responses from different retinal cell classes. Photoreceptoral function (a-wave) was quantified using a delayed-Gaussian function (Hood and Birch, 1992; Lamb and Pugh, 1992) to model ERG response at the brightest stimuli, giving two parameters, namely *RmP3* (maximal photoreceptoral output) and *S* (photoreceptoral sensitivity). ON-Bipolar cell function (b-wave) was quantified using a saturated hyperbolic function to model (Fulton and Rushton, 1978) the P2 amplitude plotted as a function of increasing luminous energies (log cd.s/m^2^) returning the maximum amplitude (*Vmax*) and sensitivity (K). Oscillatory potentials (OPs), which are small wavelets residing on the ascending limb of the b-wave, were isolated from the ERG b-wave by use of a digital band-pass filter (−3 dB at 50 to 180 Hz) and reflect amacrine cell driven inner retinal activity. The scotopic threshold response, elicited at the dimmest light levels (−5.53 to −4.90 log cd.s/m^2^), reflects RGC activity, which was measured by the peak, and peak timing of the positive component of the waveform (pSTR) (Saszik et al., 2002; Bui and Fortune, 2004).

### Optical Coherence Tomography

Following ERG recordings, the same cohort of animals underwent retinal structure imaging using spectral domain OCT (Spectralis SD-OCT, Heidelberg Engineering, Heidelberg, Germany). A drop of ocular lubricant gel (Genteal^®^ Tears, Alcon) and a coverslip was used to rehydrate and remove any opacities developed from general anesthesia. Once clear optics were achieved, excess eye gel was removed, and a single drop of Systane eye lubricant (Alcon) was applied to improve tear film optics for OCT imaging. Images were acquired with a volumetric scan pattern (8.1 mm × 8.1 mm × 1.9 mm) centered over the optic nerve head (ONH). Each volume scan consists of 121 vertical B-scans (five repeats), and each B-scan was made up of 768 A-scans. Averaged retinal layer thicknesses were extracted using an automated segmentation algorithm (Heidelberg Eye Explorer, Heyex, Heidelberg Engineering), after ensuring there were no segmentation errors, and using a pre-set annulus grid (6 mm diameter), with the inner diameter circle centered around the ONH. Retinal layers assessed included the retinal nerve fiber layer (RNFL), ganglion cell-inner plexiform layer (GC-IPL) and total retinal thickness (TRT, inner limiting membrane to Bruch’s membrane).

### Immunostaining and Quantification

Following completion of *in vivo* assessments, eyes were enucleated and fixed in 4% paraformaldehyde (all chemicals supplied by Sigma-Aldrich, Missouri, United States) diluted in 1 M phosphate buffer saline (PBS) for 1 h at room temperature. One eye was taken for cross-sectional processing and the other eye was processed for whole-mount histological analysis. Tissue from at least two mice of each cohort was determined to be unsatisfactory for histological analysis therefore *n* = 6–8 for WT and Tyro3 KO mice.

#### Retinal Cross-Section Processing

After fixation, eyes were washed once with PBS, and dissected to remove the anterior portion (cornea, iris and lens). The remaining eye cup was cryoprotected in 10, 20, and 30% sucrose overnight, embedded in optimal cutting temperature compound (Sakura Finetek, Tokyo, Japan), frozen in isopropanol, on dry ice and stored at −20°C. Retinae were cryosectioned at a thickness of 14 μm placed on SuperFrost^TM^ glass slides (Invitrogen, Thermo Fisher Scientific, Carlsbad, United States). Tissue in plane with the longitudinally sectioned optic nerve and with structural integrity at the region of interest following staining was assessed, one section per mouse was used for analysis (*n* = 6 WT, *n* = 8 Tyro3 KO).

#### Retinal Whole Mount Processing

The contralateral eye was dissected, and the anterior portion removed. Each retina was carefully isolated from the eye cup and placed in a 96 well-plate and submerged in PBS. One retina per mouse was used with four regions analyzed, two central, and two peripheral (*n* = 8 WT, *n* = 8 Tyro3 KO).

#### Immunohistological Processing for Retinal Cross-Sections

Optimal cutting compound was removed from frozen sections with 70% EtOH, rehydrated with distilled H_2_O then washed with PBS. Slides were permeabilized with 0.1% Triton X-100 in PBS. Antigen retrieval was performed with boiling sodium citrate buffer containing 0.1% Triton X-100. Slides were then washed, and tissue blocked with 10% normal donkey serum in PBS (blocking buffer). Retinae were incubated for 48 h at 4°C with primary antibodies: anti-RNA-binding protein with multiple splicing (Rbpms; 1:500, #OAGA05992, Invitrogen, Thermo Fisher Scientific) and anti-microtubule-associated protein 2 (Map2; 1:200, #NB300-213, Invitrogen, Thermo Fisher Scientific). Rbpms is a robust marker of RGCs in mice (Rodriguez et al., 2014) and Map2 immunolabels dendrites within the IPL (mostly consisting of RGC dendrites and some amacrine cell processes) (Zalis et al., 2017). Slides were washed and incubated with donkey anti-rabbit Alexa Fluor-Texas red (1:200, #711-075-152, Jackson ImmunoResearch Laboratories, Westgrove, United States) and donkey anti-chicken Alexa-488 (1:200, #703-545-155, Jackson ImmunoResearch Laboratories) diluted in blocking buffer. Retinal sections were washed, and counter-stained with Hoechst (1:1000; H1399, Invitrogen, Thermo Fisher Scientific). Slides were washed, mounted with fluorescent mounting medium (DAKO, Glostrup, Denmark) and cover-slipped. Images were acquired centrally in the cross section using a Zeiss Apotome.2 Imager.M2 (AX10; Carl Zeiss AG, Oberkochen, Germany), 40X oil objective lens (EC plan-neofluor) and Zen Blue software (2.6; Carl Zeiss AG). Ganglion cell densities were manually counted using Adobe Photoshop (Version: 19.1.6; Adobe, San Jose, United States) software. RGC densities in cross section are expressed as cells/mm ± SEM.

#### Immunohistological Processing for Retinal Whole-Mounts

Phosphate buffer saline was removed and antigen retrieval was performed in 20 mM EDTA for 1 h at 37°C, retinae were then blocked with diluent (10% normal donkey serum, 0.5% Triton X-100, 0.05% sodium azide and 0.5% dimethyl sulfoxide made in PBS). Tissue was then incubated with both rabbit-anti Rbpms antibody (as above) and chicken-anti Map2 (as above) for 72 h at room temperature on a rocker. Retinae were washed in PBS and subsequently incubated with donkey anti-rabbit Alexa Fluor-Texas red (as above) and donkey anti-chicken Alexa Fluor-488 (as above) overnight at room temperature with agitation. Retinal tissue was then washed in PBS and counter-stained with Hoechst (as above). Retinae were again washed in PBS, removed from the 96-well plate and four tension releasing cuts were created and retinae were mounted on glass microscope slides with anti-fade mounting media (as above) and a glass coverslip added. Images were acquired using a Zeiss LSM 780 confocal microscope (Carl Zeiss AG) at 40X magnification [40X/1.4 oil lens (PL-APO)]. Using Zen Black (SP5; Carl Zeiss AG) software, four images were acquired, two central (one optic disc away [∼220 μm from optic nerve head]) and two peripheral (3 optic discs away [∼880 μm from optic nerve head]) from two retinal quadrants. Images were captured as z-stacks (≤0.5 μm increments) whereby the upper limit was set by the GC layer and the lower limit to the bipolar cell layer, which was defined by the counter-stained nuclei layers. RGC counts were assessed using the spot tool in Imaris software (x64 9.5.0, Zurich, Switzerland). RGC counts are presented as averaged RGCs/mm^3^ ± SEM. Dendritic density was measured utilizing the surface tool in Imaris, surfaces were created for Map2^+ve^ segments within the grain size of 0.8 μm with a cut-off point of 0.9 μm. Surfaces then underwent manual thresholding to eliminate false positives and background staining. Map2^+ve^ surfaces were then counted by the Imaris software and expressed as total positive surfaces across % of the IPL area. ON-RGC dendrites were defined as those within the inner 0-60% of the IPL (0% was defined by the GC nuclei and 100% by the bipolar cell nuclei).

### RGC Purification and Gene Expression Analysis

RGCs were purified using immunopanning as previously described (Winzeler and Wang, 2013). Briefly, in two separate experiments (*N* = 2), post-natal day eight C57/Bl6 pups from a single litter (*n* = 5–8) were deeply anesthetized via isoflurane inhalation, decapitated and both retinae were removed. Retinae were then pooled and digested with 165 units of papain enzyme for 90 min. Tissue was then titrated, and a single cell suspension was made, RGCs were immunopanned with an anti-mouse Thy1.2 (CD90; Bio-Rad AbD Serotec, Oxford, United Kingdom) on a 10 cm petri dish. RGCs were then lysed in 700 μL of Qiazol buffer and stored at −80°C until RNA isolation. RNA was prepared using the RNeasy Plus Universal kit according to manufacturer’s instructions (Qiagen, Hilden, Germany). cDNA was transcribed from 100 ng RNA using the Applied Biosystems Taqman reverse transcription reagents (Thermo Fisher Scientific) in a total volume of 40 μL. Quantitative PCR (qPCR) reactions were performed on a Viia7 real time PCR system (Applied Biosystems, Foster City, United States) using 5 μL of cDNA with SYBR green reagents (Applied Biosystems). Primers for qPCR were as follows: 18S (fwd) 5′CGGCTACCACATCCAAGGAA3′, (rev) 5′GCTGGAATTACCGCGGCT3′ (Binder et al., 2008); Tyro3 (fwd) 5′TGGCTGAGCTGCTCCTACTTTA3′, (rev) 5′TGGGCAGTGCTGAGTTCCA3′; Rbpms (fwd) 5′ACACGAAGATGGCCAAGAAC3′, (rev) 5′ACAGTGTTGGG CAGAGGAGT3′ (Untergasser et al., 2012); Rpe65 (fwd) 5′TCATCCGCACTGATGCTTAT3′, (rev) 5′ATATTCTTGC AGGGGTCTGG3′ (Untergasser et al., 2012). Relative expression was determined using the comparative Ct method (2^–ρρ*CT*^) (Livak and Schmittgen, 2001). RNA input was normalized using the expression of 18S.

### Statistical Analyses

All statistical analyses were performed using PRISM software v.8 (GraphPad software, San Diego, United States). Single comparisons to control were made using a two-tailed Student’s *t*-test. Grouped analyses were made using repeated measures ANOVA, with Sidak’s multiple comparison test used for *post hoc* analyses. An alpha of 0.05 was used for determining statistical significance.

## Results

### Loss of Tyro3 Is Associated With a Reduction in Retinal Function

**FIGURE 1:**
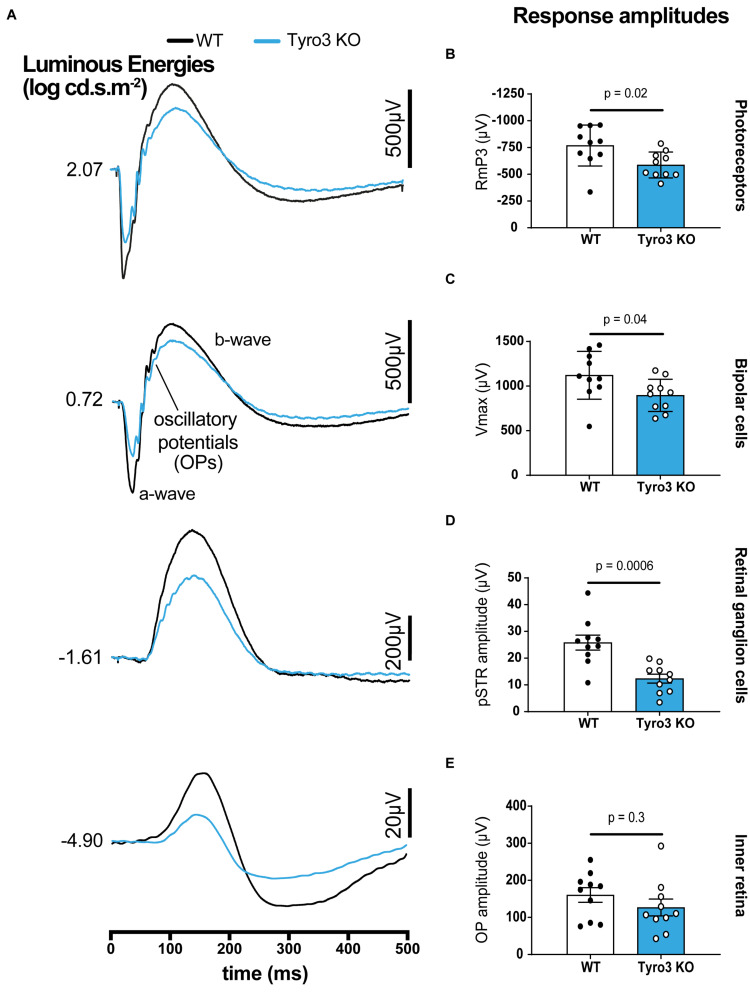
Loss of Tyro3 is associated with a reduction in retinal function. Dark-adapted full-field ERGs were performed to examine retinal function in WT and Tyro3 KO mice (*n* = 10/genotype), enabling analysis of light evoked responses from various retinal cell classes. **(A)** Average waveforms from WT mice (black) and KO mice (blue) elicited at selected light levels (bottom to top, dimmest to bright flashes, –4.90 to 2.07 log cd.s.m^– 2^). Tyro3 deficient mice showed significant reductions in amplitudes of photoreceptors (**B**; *p* = 0.02), bipolar cells (**C**; *p* = 0.04) and retinal ganglion cells (**D**; *p* = 0.006) compared with WT mice. No significant difference was observed in the amplitude of the oscillatory potentials which arise from the inner retina, reflecting amacrine cell pathways (**E**; *p* = 0.3). Results are presented as mean ± SEM; *p*-value of mean differences were calculated using a two-tailed, unpaired Student’s *t*-test.

To determine whether Tyro3 regulates the functionality of RGCs, full-field ERG recordings were undertaken on Tyro3 deficient mice and their wildtype counterparts. The latency of response was similar between Tyro3 KO mice and controls for all ERG components with the exception of a modest increase in latency of the inner retina where activity is mostly driven by amacrine cells (Figure 1A and Supplementary Figure S1; *OP peak timing*, 42.5 ± 1.5 ms vs 48 ± 1.9 ms, WT vs Tyro3 KO, respectively, *p* = 0.04). In contrast, we observed marked differences in the amplitude of the response waves of photoreceptors, bipolar cells and RGCs (Figures 1A–E). We found a 23.7% decrease in photoreceptoral function in Tyro3 KO mice compared with WT littermates (Figure 1B; *RmP3* −768.8 ± 60.5 μV vs −586.9 ± 38.1 μV, WT vs Tyro3 KO, respectively, *p* = 0.02). Similarly, the amplitude of the bipolar cell response decreased by 20.1% in the Tyro3 KO mice (Figure 1C; *Vmax* 1121.5 ± 84.9 μV vs 896.5 ± 57.2 μV, WT vs Tyro3 KO, respectively, *p* = 0.04). The RGC functional response was substantially reduced to 52.2% in Tyro3 KO mice in comparison with WT mice (Figure 1D; *pSTR amplitude* -25.8 ± 2.8 μV vs −12.4 ± 1.7 μV, WT vs Tyro3 KO, respectively, *p* = 0.0006). No significant difference was seen in peak amplitude of the inner retinal oscillatory potentials (Figure 1E; *OP peak amplitude* 160.5 ± 19.6 μV vs 126.8 ± 19.6 μV, WT vs Tyro3 KO, respectively, *p* = 0.3).

Given the vertical connectivity between photoreceptors, bipolar cells and RGCs, our observation of a decrease in function in photoreceptors could potentially account for the subsequent loss in function in downstream bipolar cells and RGCs. However, analysis of the amplitude loss in comparison to the known gain relationships between cell classes in the mouse retina (Nguyen et al., 2013) indicate that while the apparent loss in bipolar cell function can be attributed to a loss of photoreceptor function, the loss of RGC function is at least partially an independent effect (Supplementary Figure S2). To further establish a potential direct role for Tyro3 in the function of RGCs, we confirmed that, consistent with published evidence (Lindqvist et al., 2010; Rheaume et al., 2018), RGCs express *Tyro3* (Supplementary Figure S3).

### Retinal Structure Is Altered in the Absence of Tyro3

**FIGURE 2:**
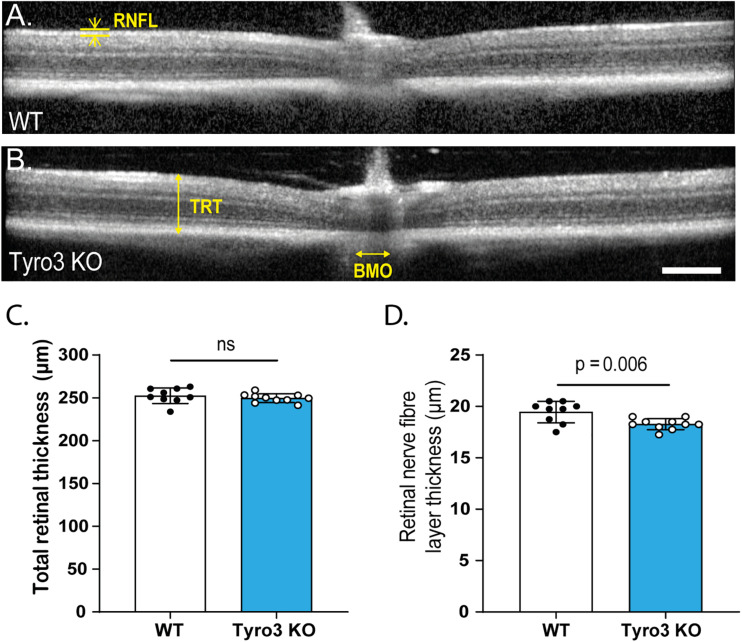
The thickness of the RNFL is reduced in the absence of Tyro3. The retinas of WT and Tyro3 KO mice were imaged *in vivo* using optical coherence tomography (OCT) (*n* = 10 mice/genotype). Representative OCT images from WT mice **(A)** and Tyro3 KO mice **(B)** are shown, with key regions identified: retinal nerve fiber layer (RNFL), total retinal thickness (TRT) and Bruch’s membrane opening (BMO). No significant difference was observed in TRT between WT and Tyro3 KO mice **(C)**. Tyro3 deficient mice displayed a significant reduction in RNFL thickness compared to WT mice (**D**; *p* = 0.006). Results are presented as mean ± SEM; *p*-value of mean differences were calculated using a two tailed Student’s *t*-test. Scale bar = 200 μm.

Given that functional changes can be the result of structural abnormalities, we investigated the retinal structure of Tyro3 KO mice using *in vivo* optical coherence tomography (OCT). The overall structure of the retina was not overtly disrupted in the absence of Tyro3 compared with WT mice (Figures 2A,B). Furthermore, total retinal thickness was not significantly reduced in the absence of Tyro3 (Figure 2D; 252.4 ± 3 μm vs 249.7 ± 1.6 μm, WT vs Tyro3 KO, respectively, *p* = 0.4). However, when the retina was stratified into specific layers, we observed a statistically significant decrease of 5.7% in the retinal nerve fiber layer (RNFL) thickness of Tyro3 deficient mice (Figure 2C; 19.4 ± 0.3 μm vs 18.3 ± 0.2 μm, WT vs Tyro3 KO, respectively, *p* = 0.006). No difference was detected in any other retinal layer (Supplementary Figure S4).

### Tyro3 Deficiency Resulted in Fewer Retinal Ganglion Cells

**FIGURE 3:**
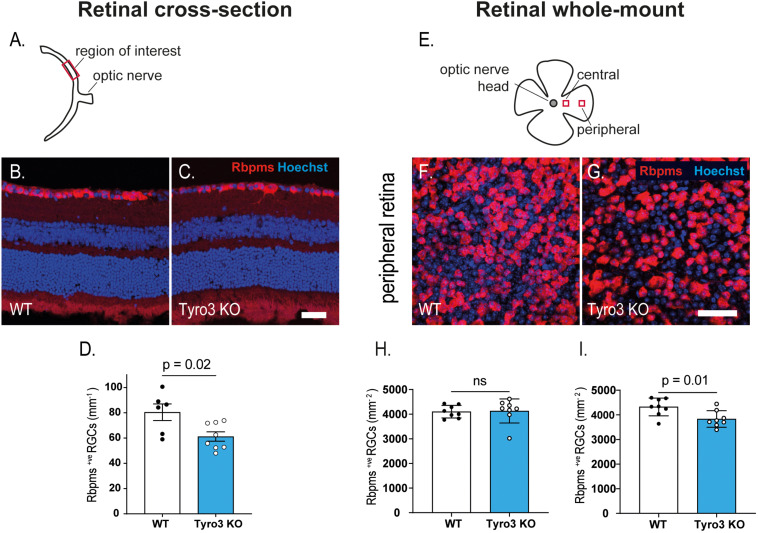
Dysregulation of retinal function is accompanied by a loss of RGCs. The density of retinal ganglion cells was determined in retinal cross-sections and in retinal flat-mounts. **(A)** Schematic of retinal cross-section identifying the region of interest. Representative images of retinal cross-sections derived from WT **(B)** and Tyro3 KO **(C)** mice stained for Rbpms (red) and Hoechst (blue) are shown. Significantly fewer Rbpms^+ve^ RGCs were observed in the Tyro3 KO (*n* = 8) mice compared with WT mice (*n* = 6) (**D**; *p* = 0.02). **(E)** Schematic representation of flat-mounted retinas identifying the two regions of interest. Representative images of flat-mounted sections derived from the peripheral region of WT **(F)**, and Tyro3 KO **(G)** mice stained for Rbpms (red) and Hoechst (blue) are shown. No difference was observed in Rbpms^+ve^ RGCs in the central retina between Tyro3 KO and WT mice **(H)**. However, significantly fewer Rbpms^+ve^ RGCs were found in the peripheral retinas derived from Tyro3 KO mice compared with WT mice (**I**; *p* = 0.01). Results are presented as mean ± SEM; *p*-value of mean differences were calculated using a two tailed Student’s *t*-test. Scale bars represent 50 μm.

As the RNFL is composed of the axons of RGCs, a reduction in the thickness of this layer suggests a loss of RGCs, which may be the driver of the observed decrease in RGC function. Initially we studied retinal cross-sections to assess the linear density of Rbpms^+ve^ RGCs (Figures 3A–C). We found that the absence of Tyro3 resulted in 24% fewer Rbpms^+ve^ RGCs than WT mice (Figure 3D; 80.5 ± 6.6 RGCs/mm v. 61.2 ± 3.7 RGCs/mm, WT vs Tyro3 KO, respectively, *p* = 0.02). As RGCs form a monolayer, cross-sectional analysis of RGC density is inherently one-dimensional. We therefore also assessed the density of RGCs using flat-mount retinas, assessing two separate regions (Figures 3E–G). We found no significant difference in the density of Rbpms^+ve^ RGCs in the central retina (Figure 3H; 4102.5 ± 90 vs 4130 ± 172.5 RGCs/mm^2^ WT vs Tyro3 KO, respectively, *p* = 0.1). However, consistent with our cross-sectional analysis, we observed 11.3% fewer RGCs in the peripheral retina of Tyro3 KO mice compared with WT mice (Figure 3I; 4321.5 ± 127.4 vs 3832.5 ± 118.6 RGCs/mm^2^, WT vs Tyro3 KO, respectively, *p* = 0.01). These results support the hypothesis that the reduction in the thickness of the RNFL in the absence of Tyro3 was due to a loss of RGCs and consequently fewer axons.

### Loss of Tyro3 Results in Fewer Dendrites Within the IPL

**FIGURE 4:**
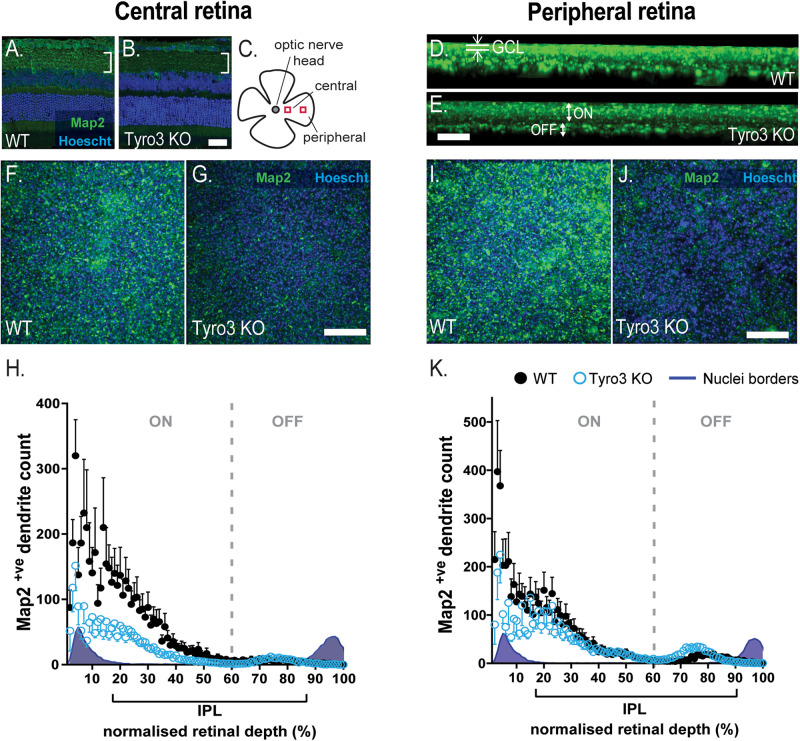
Loss of Tyro3 results in fewer RGC dendrites in the IPL. The density of dendrites within the inner plexiform layer (IPL) was assessed in the retinas of Tyro3 KO (*n* = 8) and WT mice (*n* = 6). Dendrites were visualized with fluorescent immunohistochemistry against Map2 (green). Boundaries of the inner plexiform layer (IPL) were defined by the nuclei layers counter-stained with Hoechst (blue). Cross-sectional representative images **(A,B)** and a schematic of the region of interest are shown **(C)**. Representative, z-axial images of WT and Tyro3 KO Map2 staining are shown **(D,E)**, highlighting the ganglion cell layer (GCL) and ON- OFF-dendritic regions. Flattened, transverse images from whole mount retinae from both WT and Tyro3 KO mice from the central **(F,G)** and peripheral region are shown **(I,J)**. Dendrite counts were expressed relative to the depth of the IPL, defined by the nuclei borders. Significantly fewer Map2^+ve^ dendrites were found in the IPL in the central (**H**, *p* < 0.0001) and peripheral (**K**, *p* < 0.0001) retina of Tyro3 KO mice compared to WT controls. Numerical results are presented as mean ± SEM, statistical significance was calculated using repeated measures ANOVA. Scale bars = 50 μm.

The loss of function in multiple neurons of the vertical pathway of the retina led us to hypothesize that the loss of Tyro3 may affect neuron to neuron communication, stemming from dysfunctional dendritic structure. We therefore assessed the number of Map2^+ve^ dendrites in the IPL where RGC dendrites arborize and synapse with bipolar cell terminals. We observed a global reduction in Map2 immunoreactivity in the IPL (Figures 4A–E). To quantify dendrite loss, we assessed the distribution of Map2^+ve^ dendrites from the GCL to the INL. The central retina (Figures 4F–H) and the peripheral retina (Figures 4I,J) were assessed separately.

The density of Map2^+ve^ dendrites was measured in serial z-stacks and plotted as a function of relative IPL depth, as defined by the nuclei borders of the GCL and the INL. The density of Map2^+ve^ dendrites within the central retina was observed to vary significantly with depth from the GCL [*F* (3.064, 39.83) = 13.96, *p* < 0.0001] (Figure 4H). The loss of Tyro3 also significantly reduced the density of dendrites [*F*(1, 13) = 5.114, *p* = 0.0415]. Across the whole of the IPL in the central retina, the loss of Tyro3 resulted in a 57.2% reduction in Map2^+ve^ dendrites (50.37 vs 21.58 mean dendrites in WT vs Tyro3 KO, respectively). A significant interaction between genotype and IPL depth was also observed [*F*(99,1287) = 2.762, *p* < 0.0001], although *post hoc* analyses did not identify any significant effects, likely due to loss of power from multiple analyses.

Consistent with the central retinal findings, the density of Map2^+ve^ dendrites was also observed to vary significantly with depth from the GCL in the peripheral retina [*F*(2.025, 26.33) = 20.33, *p* < 0.0001] (Figure 4K). Similarly, the loss of Tyro3 also resulted in a 26.8% reduction in the density of dendrites in the peripheral retina (54.24 vs 39.7 mean dendrites in WT vs Tyro3 KO, respectively), although the reduction was not statistically significant [*F*(1, 13) = 1.095, *p* = 0.3144]. A significant interaction effect between genotype and depth was also observed [*F*(99, 1287) = 2.158, *p* < 0.0001], however, similar to the central region, *post hoc* analyses did not identify any significant effects.

Interestingly, in both the central and peripheral retina, the greatest difference in dendrite density between WT and Tyro3 KO mice in the IPL was identified nearest to the GCL, in the sublaminae in which ON-RGCs are known to stratify and arborize (Figures 4D,E,H,K). Taken together, these data strongly support a role for Tyro3 in the elaboration or maintenance of dendrites, particularly in the central region of the retina.

## Discussion

In this study, we have identified that Tyro3 plays multiple roles within the adult mouse retina. We demonstrate that the loss of Tyro3 results in a reduction in the number of RGCs. We also establish that, independent of the loss of RGCs, Tyro3 is important in regulating aspects of RGC morphology, specifically in relation to the density of dendrites. These changes in RGC density and morphology are associated with dramatic functional deficits in the response of RGCs to light. In combination, our data identify a previously unrecognized essential role for Tyro3 in normal retinal function.

In our study, utilizing a complete developmental model of Tyro3 deletion, in adult mice, we observed a significant decrease in the amplitude response of photoreceptors, bipolar cells and RGCs. These three neurons comprise the vertical conductive pathway of the retina; given the connectivity between the neurons in this pathway, any deficit in the first order neurons would result in deficits in downstream responses. We have utilized the previously calculated “gain” relationship between ERG components (Nguyen et al., 2013) and identified that while the functional deficit in photoreceptors is sufficient to account for the reduction in bipolar cell response, the loss of function in RGCs is greater than would be predicted, suggesting that the loss of Tyro3 also has a direct effect upon RGC function.

What might be the mechanism by which the loss of Tyro3 results in photoreceptor dysfunction? It has long been known that disruption of the Tyro3-related receptor, Mertk, results in degeneration of photoreceptors and ultimately blindness, due to the failure of RPE cells to appropriately phagocytose the outer discs of the photoreceptors (D’Cruz et al., 2000). More recently, it was shown that Tyro3 is a genetic modifier of this process (Vollrath et al., 2015). Although the effect of Tyro3 deletion on retinal structure had been investigated in these studies, to our knowledge, this study is the first to assess the function of the retina in these mice. Specifically, we did not find a change in outer retinal thickness in the Tyro3 KO mouse, as might be expected with aberrant RPE phagocytosis of photoreceptor outer segments. It therefore seems probable that the loss of Tyro3 in the RPE may result in subtle deficits which cannot be identified solely through structural analysis. Indeed, our data confirm previously published evidence that Tyro3 deletion does not result in major changes to retinal structure (Prasad et al., 2006). The alternative hypothesis of course is that the loss of Tyro3 has direct effects upon photoreceptors, potentially through ionic channels that influence the dark current. However, we find no light sensitivity deficit in photoreceptors, suggesting the G-protein cascade is not affected. To formally determine the cause of photoreceptor dysfunction, cell specific deletion of Tyro3 or immunohistochemical quantification of the photoreceptors and RPE would be required.

In addition to the functional deficit in photoreceptors, we identified a second, independent disruption of RGC function. As it was not measured, we cannot rule out that the decrease in RGC function was influenced by an increase in intraocular pressure within the Tyro3 deficient mice (Bui et al., 2013). However, we believe the decrease in RGC function in the absence of Tyro3 is direct as it was accompanied by a loss of RGCs, a phenotype which has previously been shown to correlate with decreased RGC response amplitude (Hare et al., 2001). Surprisingly, the loss of RGCs was moderate and not observed throughout the GCL but confined to the peripheral retina. We observed that Tyro3 deficient mice had 11% fewer RGCs in the peripheral retina but no loss of RGCs in the central retina. The question then arises as to whether this moderate RGC loss would result in such a large reduction in the ERG amplitude. Certainly, in other models, moderate RGC loss can lead to a reduction in ERG amplitude (Liu et al., 2014), but not to the extent we observed in this study. It therefore seems probable that the functional deficit in the RGCs pSTR we have observed in our Tyro3 deficient mice is only partially explained by the loss of RGCs.

In addition to the loss of RGCs, we have identified that Tyro3 is a critical regulator of RGC dendrite morphology, particularly in the central retina. Tyro3 deficient animals had substantially fewer RGC dendrites than WT mice, with the largest differences observed in the IPL sublaminae nearest the GCL, where the dendrites of ON-RGCs are known to stratify. It is striking that the largest loss of dendrites was in the central retina which showed no detectable reduction in RGC densities, indicating that the dendritic phenotype cannot be attributed to cell loss.

A role for Tyro3 in neuronal dendrites has previously been proposed. Prieto et al. (2007) have shown that Tyro3 is enriched in the dendrites of glutamatergic neurons of the hippocampus, and suggested Tyro3 may play a role in synaptic plasticity (Prieto et al., 2007). Although we have not directly addressed the mechanism by which dendrite density is reduced in the absence of Tyro3, the functional consequences are profound. As discussed above, the loss of RGCs is unlikely, in isolation, to account for the extent of RGC dysfunction detected by ERGs. It is reasonable to suggest that the reduction in the number of dendrites, which was greater than 50% in the central retina, contributes substantially to the disruption of retinal function.

Notably, we have identified regional differences in both RGC survival and dendritic loss. In the RPE, Tyro3 has been previously shown to have a gradient of expression from higher expression in the central retina to lower in the periphery (Vollrath et al., 2015). Such expression gradients could also extend to other regions of the retina, including the GCL. The existence of a gradient, however, cannot completely account for the difference between the moderate loss of RGCs which was confined to the peripheral retina, and the reduction in dendrites, which although more substantial in the central retina, does extend to the peripheral retina. One possibility is that Axl, another TAM receptor, may be able to compensate for Tyro3 in supporting RGC survival, but not in promoting dendrite morphology. It has been shown previously that double Axl and Tyro3 knockout mice have disrupted gonadotropin-releasing hormone, neuron survival (Pierce et al., 2008), and that Axl was expressed by RGCs (Lindqvist et al., 2010). In contrast to Tyro3, however, Axl is not known to be expressed in the dendrites of neurons, suggesting the possibility that Axl could compensate for Tyro3 in supporting RGC survival, but not in promoting dendrite density. Whether or not Axl can in this instance compensate for the loss of Tyro3 and, moreover, whether Axl itself is subject to regional differences in expression within the retina, will require further investigation to formally determine.

How could Tyro3 be affecting RGC dendrites? Another receptor tyrosine kinase, TrkB, is critical for activity dependent lamina pruning of the IPL during development (Liu et al., 2007; Grishanin et al., 2008). Liu et al. (2007) showed that TrkB is crucial for activity dependent ON-RGC dendrite establishment, and in the absence of visual stimulation, the addition of BDNF, the cognate ligand of TrkB, can rescue IPL stratification. Tyro3 could potentially be acting in a similar manner to TrkB, with a role in activity dependent ON-RGC establishment within the IPL. In addition to a role in enhancing RGC survival, Tyro3 could also be acting in an activity dependent, ligand-induced signal transducing manner to regulate dendritic stratification in the IPL. This is interesting as it could mean that different subpopulations have been lost or enriched in the Tyro3 deficient mice, ultimately with implications for function. Whether Tyro3 has a role in just dendrites of the retina, or also more extensively within the CNS, remains to be determined. As Tyro3 has previously been shown to be expressed in the dendrites of other neurons (Prieto et al., 2007), it seems likely that this affect would extend beyond the retina, and the dendritic density in high Tyro3 expressing areas of the brain would also be dysregulated.

Moreover, the identification of a loss of RGCs and a change in dendritic morphology may be important in interpreting our prior findings, whereby we have previously identified that Tyro3 deficient mice had a transient delay in myelination, but a chronic reduction in myelin thickness in both the optic nerve and corpus callosum (Akkermann et al., 2017; Blades et al., 2018). As both neurons and oligodendrocytes express Tyro3, we could not formally attribute this myelin phenotype to a particular cell type. However, a decrease in dendritic density and a reduction in RGC functional output could plausibly result in decreased activity dependent myelination and contribute to our previously reported thinner myelin phenotype.

An important future extension to this study will be to understand which of the TAM ligands are essential in driving this process. Tyro3 is activated by both Gas6 and Pros1, and both are expressed in RGCs (Lindqvist et al., 2010). Furthermore, it has been established that both ligands function in the RPE to drive normal phagocytosis of photoreceptor outer discs (Burstyn-Cohen et al., 2012). In addition, both ligands are neuroprotective in multiple degenerative models (Yagami et al., 2002; Liu et al., 2003; Zhong et al., 2010), raising the possibility that signaling via the TAM receptors could provide a novel target for therapeutic development in eye diseases such as glaucoma. Future studies examining the effect of Tyro3 deficiency upon outcome in eye disease models will be critical.

We show that the loss of Tyro3 results in a reduction in photoreceptor function and an independent disruption of RGC function. The disruption in normal RGC function is associated with a loss of a subset of RGCs in the mouse retina. In addition, we have identified a novel role for Tyro3 in either the establishment or maintenance of RGCs dendrites. Investigating the pathways and mechanisms in which Tyro3 regulates RGC survival and morphology could inform new therapeutic approaches in retinal diseases.

## Data Availability Statement

All datasets presented in this study are included in the article/Supplementary Material.

## Ethics Statement

The animal study was reviewed and approved by The Florey Institute of Neuroscience and Mental Health Animal Ethics Committee.

## Author Contributions

FB, MB, VW, CN, BB, and TK contributed to the conception or design of the work, data analysis and interpretation, drafting the article, critical revision of the article, and final approval of the version to be published. FB, MB, VW, and CN contributed to data collection. All authors contributed to the article and approved the submitted version.

## Conflict of Interest

The authors declare that the research was conducted in the absence of any commercial or financial relationships that could be construed as a potential conflict of interest.

## Supplementary Material

FIGURE S1 Sensitivity and timing of electroretinogram components in Tyro3 KO and wildtype mice. Dark-adapted full-field ERGs were performed to examine retinal function in WT and Tyro3 KO mice (*n* = 10/genotype), enabling sensitivity and timing of various retinal cell classes to be quantified. Tyro3 deficient mice showed no significant reductions in the photoreceptor sensitivity **(A)** and bipolar cell sensitivity **(B)** to light compared to WT mice. The peak time of the retinal GC driven positive scotopic threshold response **(C)** was similar to the WT group. In contrast, a small but significant decrease in response timing of the oscillatory potentials, reflecting inhibitory pathways (amacrine cells) was observed in the retina of Tyro3 KO mice compared to WT controls (D; *p* = 0.04). Results are presented as mean ± SEM; *p*-value of mean differences were calculated using a two-tailed, unpaired Student’s *t*-test.

FIGURE S2 The reduction in RGC function is independent of the reduction in photoreceptor function. In order to determine whether the loss of bipolar cell function and RGC function as assessed by ERG component amplitude could be attributed to the loss of photoreceptor function, we used previously determined gain relationship calculations between components in C57Bl/6 mice (Nguyen et al., 2013). The reduction in function of cell classes in Tyro3 mice is shown relative (% ± SEM) to the WT group. Gray boxes represent the 95% confidence interval (CI) of the gain relationship between photoreceptors and downstream cell types. Functional losses are considered independent of upstream functional losses if they fall outside the CI.

FIGURE S3 Tyro3 mRNA is expressed in RGCs. The expression of the Tyro3 gene in purified primary mouse RGCs was quantified using reverse transcription-quantitative polymerase chain reaction (qPCR). **(A)** The purity of the RGC preparation was assessed using the expression of the key RGC gene (Rbpms) in purified cells derived from WT mice (*n* = 5–8, *N* = 2) compared with whole retina. A greater than four-fold enrichment of Rbpms mRNA in purified cells was observed, although some contamination with RPE cells was also detected. **(B)** Tyro3 mRNA expression is enriched in purified RGCs compared with the whole retina.

FIGURE S4 Optical coherence tomography. The retinas of WT and Tyro3 KO mice were imaged using optical coherence tomography (OCT) (*n* = 10 mice/genotype) **(A)** ganglion cell-inner plexiform layer (GC-IPL), **(B)** the inner nuclear layer (INL), **(C)** the outer plexiform layer (OPL), **(D)** the outer nuclear layer (ONL), **(E)** the outer segment of the photoreceptor layer (OUTER), and **(F)** the retinal pigment epithelium (RPE) were quantified. No significant differences were observed in the thicknesses of the layers described **(A–F)**. Results are presented as mean ± SEM; *p*-value of mean differences were calculated using a two tailed Student’s *t*-test.
