# Supplementary figures and images for: Tyro3 Contributes to Retinal Ganglion Cell Function, Survival and Dendritic Density in the Mouse Retina
Source: Front Neurosci. 2020 Aug 14;14:840. doi: 10.3389/fnins.2020.00840 (PMC7457004; doi:10.3389/fnins.2020.00840)

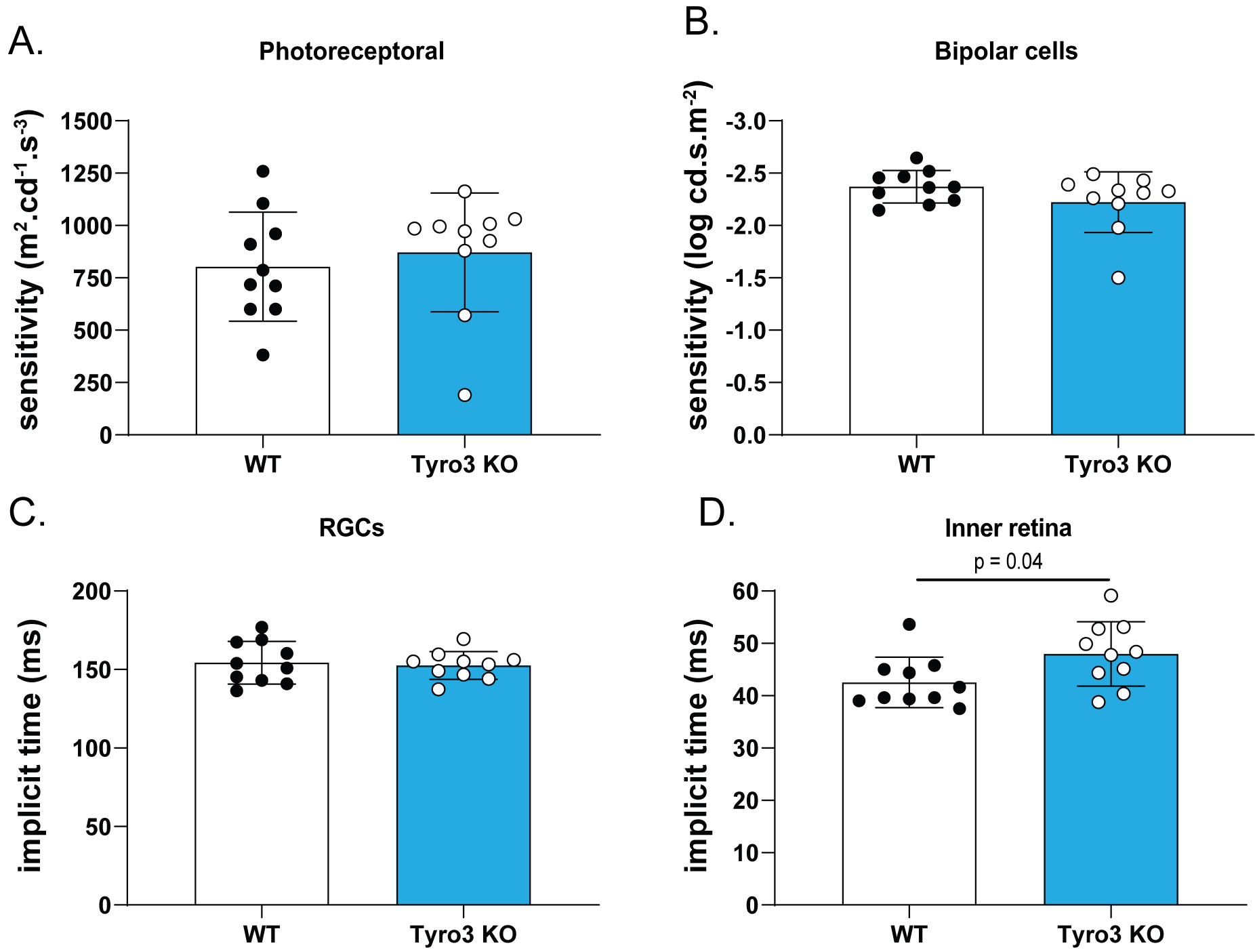

Supplement: FIGURE S1 — Sensitivity and timing of electroretinogram components in Tyro3 KO and wildtype mice. Dark-adapted full-field ERGs were performed to examine retinal function in WT and Tyro3 KO mice (n = 10/genotype), enabling sensitivity and timing of various retinal cell classes to be quantified. Tyro3 deficient mice showed no significant reductions in the photoreceptor sensitivity (A) and bipolar cell sensitivity (B) to light compared to WT mice. The peak time of the retinal GC driven positive scotopic threshold response (C) was similar to the WT group. In contrast, a small but significant decrease in response timing of the oscillatory potentials, reflecting inhibitory pathways (amacrine cells) was observed in the retina of Tyro3 KO mice compared to WT controls (D; p = 0.04). Results are presented as mean ± SEM; p-value of mean differences were calculated using a two-tailed, unpaired Student’s t-test. [file Image_1.TIF]

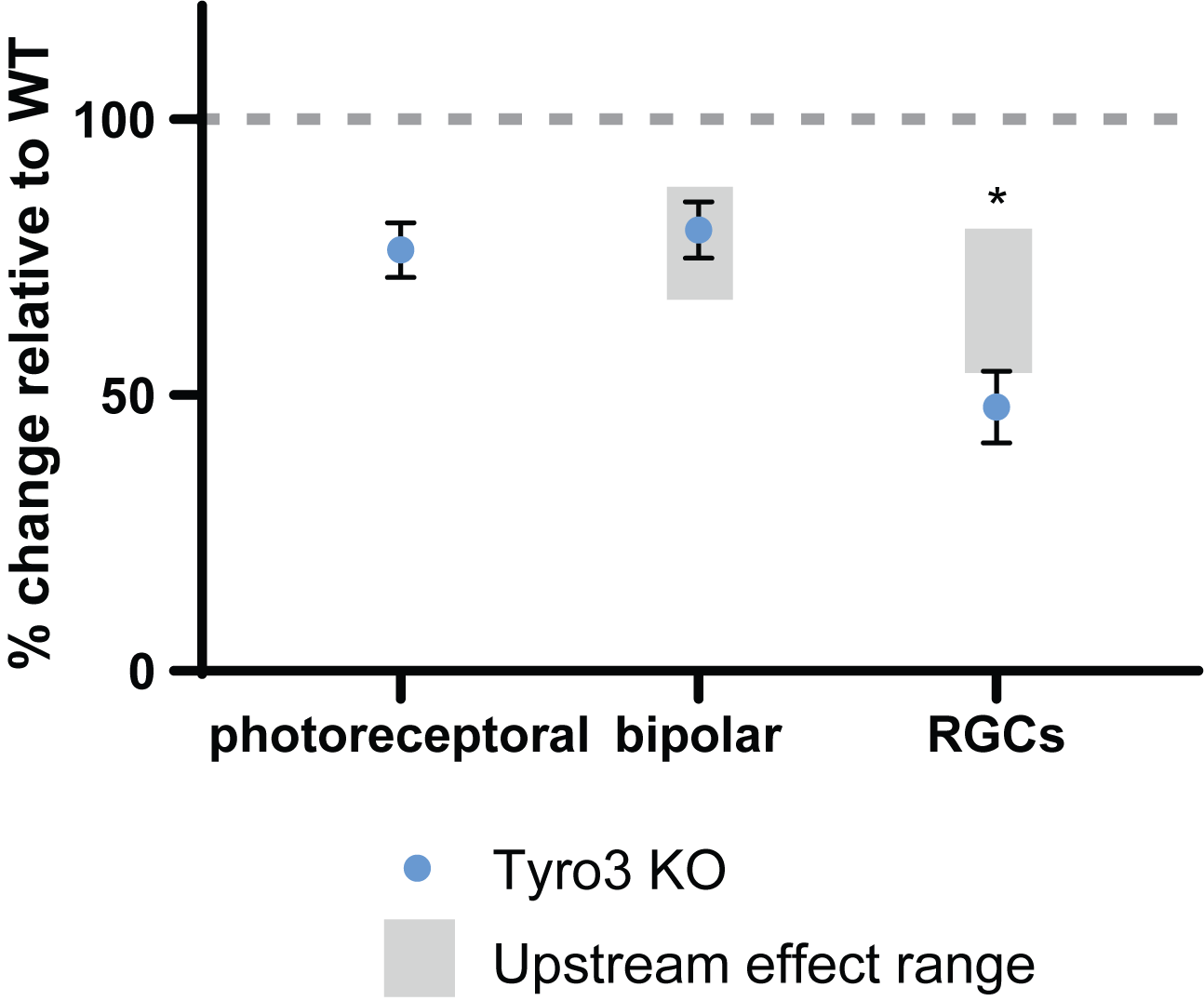

Supplement: FIGURE S2 — The reduction in RGC function is independent of the reduction in photoreceptor function. In order to determine whether the loss of bipolar cell function and RGC function as assessed by ERG component amplitude could be attributed to the loss of photoreceptor function, we used previously determined gain relationship calculations between components in C57Bl/6 mice (Nguyen et al., 2013). The reduction in function of cell classes in Tyro3 mice is shown relative (% ± SEM) to the WT group. Gray boxes represent the 95% confidence interval (CI) of the gain relationship between photoreceptors and downstream cell types. Functional losses are considered independent of upstream functional losses if they fall outside the CI. [file Image_2.TIF]

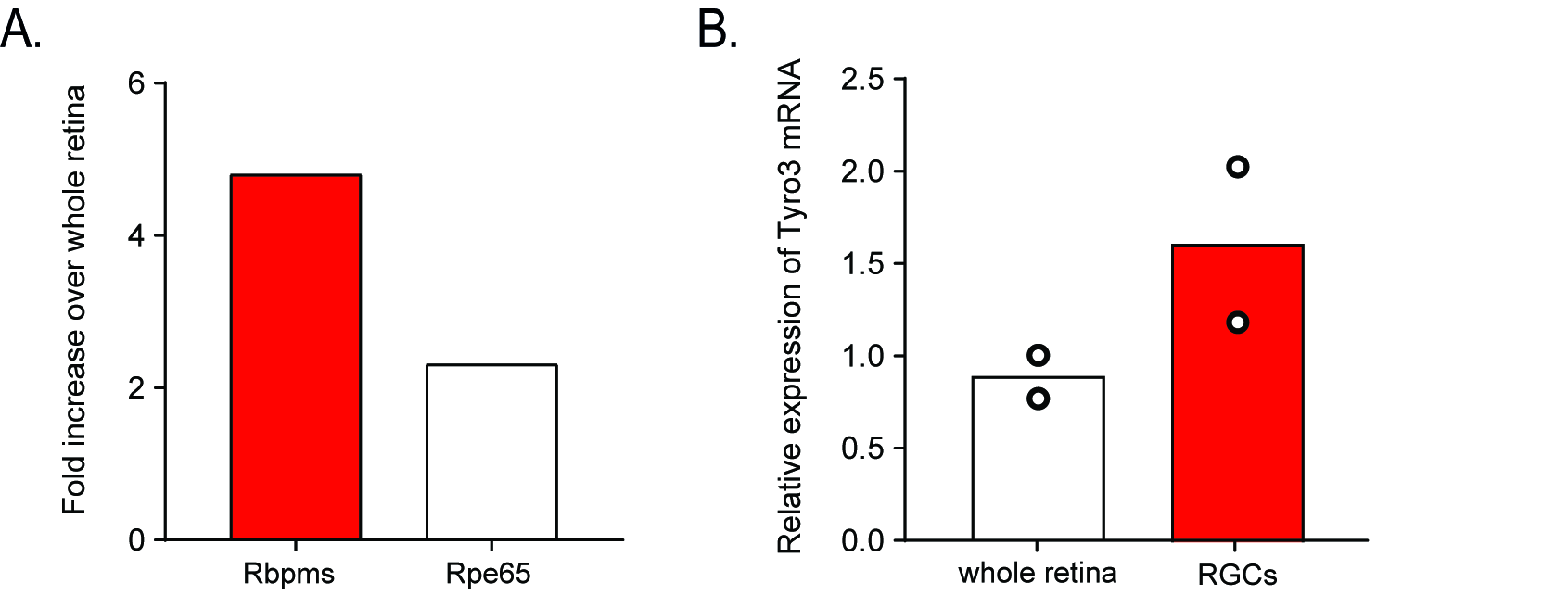

Supplement: FIGURE S3 — Tyro3 mRNA is expressed in RGCs. The expression of the Tyro3 gene in purified primary mouse RGCs was quantified using reverse transcription-quantitative polymerase chain reaction (qPCR). (A) The purity of the RGC preparation was assessed using the expression of the key RGC gene (Rbpms) in purified cells derived from WT mice (n = 5–8, N = 2) compared with whole retina. A greater than four-fold enrichment of Rbpms mRNA in purified cells was observed, although some contamination with RPE cells was also detected. (B) Tyro3 mRNA expression is enriched in purified RGCs compared with the whole retina. [file Image_3.TIF]

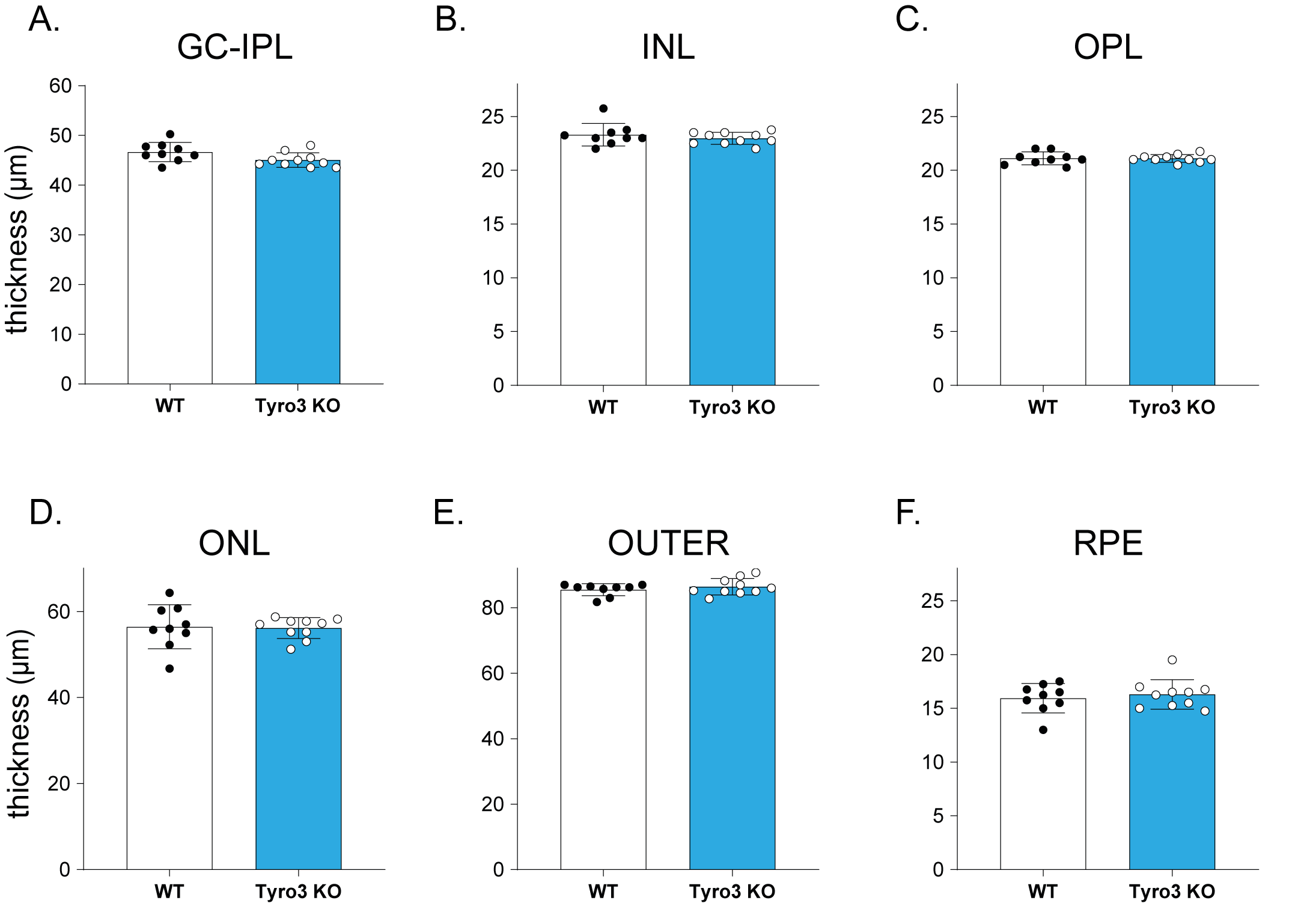

Supplement: FIGURE S4 — Optical coherence tomography. The retinas of WT and Tyro3 KO mice were imaged using optical coherence tomography (OCT) (n = 10 mice/genotype) (A) ganglion cell-inner plexiform layer (GC-IPL), (B) the inner nuclear layer (INL), (C) the outer plexiform layer (OPL), (D) the outer nuclear layer (ONL), (E) the outer segment of the photoreceptor layer (OUTER), and (F) the retinal pigment epithelium (RPE) were quantified. No significant differences were observed in the thicknesses of the layers described (A–F). Results are presented as mean ± SEM; p-value of mean differences were calculated using a two tailed Student’s t-test. [file Image_4.TIF]
